# Supplementary material for: Losing Control: Sleep Deprivation Impairs the Suppression of Unwanted Thoughts
Source: Clin Psychol Sci. 2020 Oct 15;9(1):97–113. doi: 10.1177/2167702620951511 (PMC7820573; doi:10.1177/2167702620951511)
Supplement: Harrington_Supplemental_Material – Supplemental material for Losing Control: Sleep Deprivation Impairs the Suppression of Unwanted Thoughts [file Harrington_Supplemental_Material.pdf]

Losing Control: Sleep Deprivation Impairs the Suppression of Unwanted Thoughts

Recognition Task S1

At the end of the experiment, memory for all of the face-scene pairs was tested using a recognition task. This task was included to ensure that participants had retained knowledge of the face-scene pairs across the overnight interval.

On each trial, participants viewed a single face, together with two scenes: one that was paired with the face and another which featured in the experiment but was not paired with this particular face. Participants were instructed to indicate which scene was paired with the face via key press within 5 s. We asked participants to make this response as quickly and accurately as possible. The trial terminated once a response had been provided or the time limit expired, before the next trial began.

Recognition accuracy was calculated as the proportion of face-scene associations that were correctly identified. Data were analysed using a 3 (TNT Condition: ‘Baseline’/’No-Think’/’Think’) x 2 (Valence: Negative/Neutral) x 2 (Group: Sleep/Sleep Deprivation) mixed ANOVA.

Recognition accuracy was very high in both groups [sleep group: M = 97.70%, SEM = 0.54%; sleep deprivation group: M = 96.32%, SEM = 0.71%], suggesting that knowledge of the face-scene pairs was well-retained across the overnight interval. There was no statistically significant difference in recognition performance between groups [$F(1,57) = 2.37$, $p = .129$], and there was no significant main effect of TNT Condition [$F(1.80,102.54) = 2.69$, $p = .078$, *Greenhouse-Geisser corrected*] or Valence [$F(1,57) = 0.36$, $p = .551$], and there were no significant interactions [all $p > .05$].

Losing Control: Sleep Deprivation Impairs the Suppression of Unwanted Thoughts

Methodological Details S1

Electrodermal activity was recorded using a BIOPAC MP36R data acquisition system and AcqKnowledge (ACQ) 4.4.1 software (sampling rate = 2KHz). During the affect evaluation tasks, E-Prime-triggered square pulse outputs were transmitted to the MP36R unit via a BIOPAC STP35A interface enabling precise alignment of each stimulus onset to the skin conductance response (SCR) data. Two BIOPAC EL507 disposable adhesive electrodes were attached to the fingertips of the index and middle fingers of the non-dominant hand. The data were imported and preprocessed using PsPM (version 4.0.2; Bach & Friston, 2013).

A unidirectional first-order Butterworth high-pass filter with cut-off frequency 0.05 Hz was used to filter the data to account for the change of baseline activity during the duration of the recording sessions. The time series averaged over corresponding trials for each experimental condition (e.g. 'No-Think', negative valence) were then extracted, for each subject, for each of the two sessions (pre-TNT and post-TNT; TNT = Think/No-Think). An average 'session-specific' skin conductance level (SCL) was computed for each subject for each session. SCL was the skin conductance value measured for the first second after the presentation of the stimuli, averaged across all conditions. This first 1 s period after stimulus presentation is widely considered to be the 'latency' period for event-related evoked SCRs (Bach, Flandin, Friston, & Dolan, 2010; Braithwaite, Watson, Robert, & Mickey, 2013; Lim et al., 1997). This 'baseline' SCL was then subtracted from the rest of the measured skin conductance activity, which was deemed to belong to a canonical evoked SCR, taken for the entirety of the time that the stimulus was presented on screen during a trial, after excluding the first second (i.e. 5.5 seconds). The area under the curve for each condition was then computed for each subject, for each of the two sessions. This is akin to an analysis approach shown previously for spontaneous skin conductance fluctuations, except here we have used it to analytically quantify event-related evoked SCRs (Bach, Friston, & Dolan, 2010). The average SCRs elicited by scenes in each TNT condition and valence category were used to calculate the difference in SCRs across sessions (dSCR; SCR post-TNT – SCR pre-TNT). The same analysis pipeline was used for

both the sleep and the sleep deprivation groups. Data from 2 participants were unavailable due to technical issues (sleep group *n*=1; sleep deprivation group *n*=1). Furthermore, we excluded data from 3 participants in the sleep group who were SCR non-responders.

References

Bach, D. R., Flandin, G., Friston, K. J., & Dolan, R. J. (2010). Modelling event-related skin conductance responses. *International Journal of Psychophysiology*, *75*(3), 349–356. https://doi.org/10.1016/j.ijpsycho.2010.01.005

Bach, D. R., & Friston, K. J. (2013). Model-based analysis of skin conductance responses: Towards causal models in psychophysiology. *Psychophysiology*, *50*(1), 15–22. https://doi.org/10.1111/j.1469-8986.2012.01483.x

Bach, D. R., Friston, K. J., & Dolan, R. J. (2010). Analytic measures for quantification of arousal from spontaneous skin conductance fluctuations. *International Journal of Psychophysiology*, *76*(1), 52–55. https://doi.org/10.1016/j.ijpsycho.2010.01.011

Braithwaite, J. J., Watson, D. G., Robert, J., & Mickey, R. (2013). *A Guide for Analysing Electrodermal Activity (EDA) & Skin Conductance Responses (SCRs) for Psychological Experiments*. *Selective Attention & Awareness Laboratory (SAAL) Behavioural Brain Sciences Centre, University of Birmingham, UK*.

Lim, C. L., Rennie, C., Barry, R. J., Bahramali, H., Lazzaro, I., Manor, B., & Gordon, E. (1997). Decomposing skin conductance into tonic and phasic components. *International Journal of Psychophysiology*, *25*(2), 97–109. https://doi.org/10.1016/S0167-8760(96)00713-1

Losing Control: Sleep Deprivation Impairs the Suppression of Unwanted Thoughts

Methodological Details S2

Electrocardiography (ECG) was recorded using a BIOPAC MP36R data acquisition system and AcqKnowledge (ACQ) 4.4.1 software (sampling rate = 2KHz). Three BIOPAC EL503 ECG electrodes were attached to the midline of the left and right clavicle and the lower left rib. ECG was recorded for eight successive minutes. The first 2 min and last 1 min of each recording was discarded, and heart rate variability (HRV) was calculated for the remaining five successive minutes. The ECG signal was analysed offline using ACQ and Kubios Standard 3.0.2 software.

R-peaks were automatically detected using ACQ's QRS detection algorithm and visually inspected for accuracy. Peaks that the algorithm missed were inserted manually. The interbeat-interval time series was then imported to Kubios for analysis. To obtain frequency-domain-specific indices of HRV, we used autoregressive estimates of low-frequency (0.04-0.15 ms^2/Hz) and high-frequency (0.15-0.40 ms^2/Hz) power. Autoregressive algorithms are generally preferable to Fourier transform based algorithms for spectral analysis of HRV (Thayer, Hansen, & Johnson, 2008), partly because they have better spectrum resolution when using short data frames (Miranda et al., 2012). In keeping with previous research (Gillie, Vasey, & Thayer, 2014; Park, Vasey, Van Bavel, & Thayer, 2014), values of low-frequency HRV (LF-HRV) and high-frequency HRV (HF-HRV) were transformed logarithmically (base 10). One participant in the sleep group exhibited atypical ECG patterns, and was thus removed from HRV analyses.

References

Gillie, B. L., Vasey, M. W., & Thayer, J. F. (2014). Heart Rate Variability Predicts Control Over Memory Retrieval. *Psychological Science*, *25*(2), 458–465. https://doi.org/10.1177/0956797613508789

Laborde, S., Mosley, E., & Thayer, J. F. (2017). Heart rate variability and cardiac vagal tone in psychophysiological research - Recommendations for experiment planning, data analysis, and data reporting. *Frontiers in Psychology*, *8*(FEB), 1–

18. https://doi.org/10.3389/fpsyg.2017.00213

Miranda, E., Lima, M., Anna, S., Varej, R., Gonc, C. P., Morra, E. A., … Mill, G. (2012). Spectral analysis of heart rate variability with the autoregressive method: What model order to choose?, *42*, 164–170. https://doi.org/10.1016/j.compbiomed.2011.11.004

Park, G., Vasey, M. W., Van Bavel, J. J., & Thayer, J. F. (2014). When tonic cardiac vagal tone predicts changes in phasic vagal tone: The role of fear and perceptual load. *Psychophysiology*, *51*(5), 419–426. https://doi.org/10.1111/psyp.12186

Thayer, J. F., Hansen, A. L., & Johnson, B. H. (2008). Non-invasive assessment of autonomic influences on the heart: Impedance cardiography and heart rate variability. In *Handbook of Physiological Research Methods in Health Psychology* (pp. 183–209). Newbury Park, CA: Sage Publications.

Losing Control: Sleep Deprivation Impairs the Suppression of Unwanted Thoughts

Methodological Details S3

Sleep monitoring was carried out using an Embla N7000 polysomnography (PSG) system (Embla Systems, Broomfield, CO, USA). Gold-plated electrodes were attached using EC2 electrode cream after the scalp was cleaned with NuPrep exfoliating agent. Scalp electrodes were attached at eight standard locations according to the international 10-20 system (Homan, Herman, & Purdy, 1987): F3, F4, C3, C4, P3, P4, O1, and O2, each referenced to the contralateral mastoid (A1 or A2). Left and right electrooculogram, left, right and upper electromyogram, and a ground electrode (forehead) were also attached. All electrodes were verified to have a connection impedance of < 5 kΩ. All signals were digitally sampled at a rate of 200 Hz.

Sleep data was divided into 30 s epochs and scored as wakefulness, N1 sleep, N2 sleep, slow-wave sleep (SWS) or rapid eye movement (REM) sleep according to standardised criteria (Iber, Ancoli-Israel, Chesson, & Quan, 2007), using RemLogic 3.4. PSG data was unavailable for 4 participants due to reference electrodes becoming detached during the night.

References

Homan, R. W., Herman, J., & Purdy, P. (1987). Cerebral location of international 10-20 system electrode placement. *Electroencephalography and Clinical Neurophysiology*, *66*, 376–382. https://doi.org/10.1016/0013-4694(87)90206-9

Iber, C., Ancoli-Israel, S., Chesson, A., & Quan, S. F. (2007). *The AASM manual for the scoring of sleep and associated events rules, terminology, and technical specifications*. Westchester, IL: American Academy of Sleep Medicine.

Losing Control: Sleep Deprivation Impairs the Suppression of Unwanted Thoughts

Table S1

Table S1. Sleep stage data

N1 (min; %)	8.02 ± 1.07	1.96 ± 0.26
N2 (min; %)	218.14 ± 6.76	53.56 ± 1.25
SWS (min; %)	107.34 ± 3.85	26.47 ± 0.93
REM (min; %)	73.00 ± 3.87	18.00 ± 0.95
TST (min)	406.50 ± 6.78	-
Sleep efficiency (%)	97.26 ± 0.49	-

Data presented as mean ± SEM. Sleep efficiency refers to the proportion of time spent asleep between sleep onset and final awakening. Abbreviations: N1, N2, stages of non-REM sleep; SWS, slow-wave sleep; REM, rapid eye movement sleep; TST, total sleep time.
